# Pan-Cancer Analysis Reveals the Signature of TMC Family of Genes as a Promising Biomarker for Prognosis and Immunotherapeutic Response

**DOI:** 10.3389/fimmu.2021.715508

**Published:** 2021-11-09

**Authors:** Jing Song, Yongyao Tang, Xiaoyong Luo, Xinpeng Shi, Fangzhou Song, Longke Ran

**Affiliations:** ^1^ Department of Bioinformatics, The Basic Medical School of Chongqing Medical University, Chongqing, China; ^2^ Molecular and Tumor Research Center, Chongqing Medical University, Chongqing, China; ^3^ Department of Oncology, The Affiliated Luoyang Central Hospital of Zhengzhou University, Luoyang, China; ^4^ Forensic Laboratory, The Basic Medical School of Chongqing Medical University, Chongqing, China

**Keywords:** transmembrane channel-like proteins, multi-omics, pan-cancer analysis, biomarker, immune-infiltrating, immunotherapy

## Abstract

Transmembrane Channel-like (TMC) genes are critical in the carcinogenesis, proliferation, and cell cycle of human cancers. However, the multi-omics features of TMCs and their role in the prognosis and immunotherapeutic response of human cancer have not been explored. We discovered that TMCs 4-8 were commonly deregulated and correlated with patient survival in a variety of cancers. For example, TMC5 and TMC8 were correlated with the relapse and overall survival rates of breast cancer and skin melanoma, respectively. These results were validated by multiple independent cohorts. TMCs were regulated by DNA methylation and somatic alterations, such as TMC5 amplification in breast cancer (523/1062, 49.2%). Six algorithms concordantly uncovered the critical role of TMCs in the tumor microenvironment, potentially regulating immune cell toxicity and lymphocytes infiltration. Moreover, TMCs 4-8 were correlated with tumor mutation burden and expression of PD-1/PD-L1/CTLA4 in 33 cancers. Thus, we established an immunotherapy response prediction (IRP) score based on the signature of TMCs 4-8. Patients with higher IRP scores showed higher immunotherapeutic responses in five cohorts of skin melanoma (area under curve [AUC] = 0.90 in the training cohort, AUCs range from 0.70 to 0.83 in the validation cohorts). Together, our study highlights the great potential of TMCs as biomarkers for prognosis and immunotherapeutic response, which can pave the way for further investigation of the tumor-infiltrating mechanisms and therapeutic potentials of TMCs in cancer.

## Introduction

Cancer is a disease in cells with multi-omics dysregulations, such as genetic alterations, differential DNA methylations, and transcriptomic and metabolic disorders (1). Advances in high-throughput sequencing and bioinformatic technologies over the past decades have allowed millions of omics-level alterations to be detected, and made it possible to systematically study their roles in tumorigenesis and progression (2). However, most of the multi-omics alterations are not well understood. For example, millions of genetic mutations were detected in different cancer types, while their roles in immunotherapeutic response and tumor microenvironment were largely unknown, and their clinical potentials were not fully studied. Moreover, the genes altered across different tumor types and individual tumor samples vary considerably (3). Thus, a complete understanding of the omics-level alterations in different cancer types is essential to identify novel potential therapeutic targets and vulnerabilities.

A comprehensive analysis for the expression profile of immune checkpoint genes at a pan-cancer level identified immune checkpoint blockade based therapy-related biomarkers, which indicated that gene classes with the same functions may serve as a molecular signature (4). Transmembrane channel-like (TMC) proteins are a gene family of evolutionarily conserved ion channel-like membrane proteins. Based on the amino-acid sequence similarities, TMC channels form three subfamilies: TMCs 1–3 (subfamily 1), TMCs 5 and 6 (subfamily 2), and TMCs 4, 7, and 8 (subfamily 3) (5, 6). In the past two decades, TMCs were reported to play an important role in hearing loss (7), fatty liver disease (8), and cancer (9). TMC1 and TMC2 were studied best because of their essential role in auditory transduction (10, 11). The functional research of TMCs 3-8 in human cancers is largely unexplored. In recent years, evidence has indicated that TMC6 (also known as EVER1) and TMC8 (also known as EVER2) play a role in cervical cancer (12) and squamous cell carcinoma (9). Notably, we recently found that TMC5 promotes prostate cancer cell proliferation through cell cycle regulation (13). Thus, TMCs are critical regulators in diverse human cancers, and systematic investigation of TMCs in cancers is necessary and valuable for a better understanding of their roles in cancer development and their clinical therapeutic potentials.

**Figure 1:**
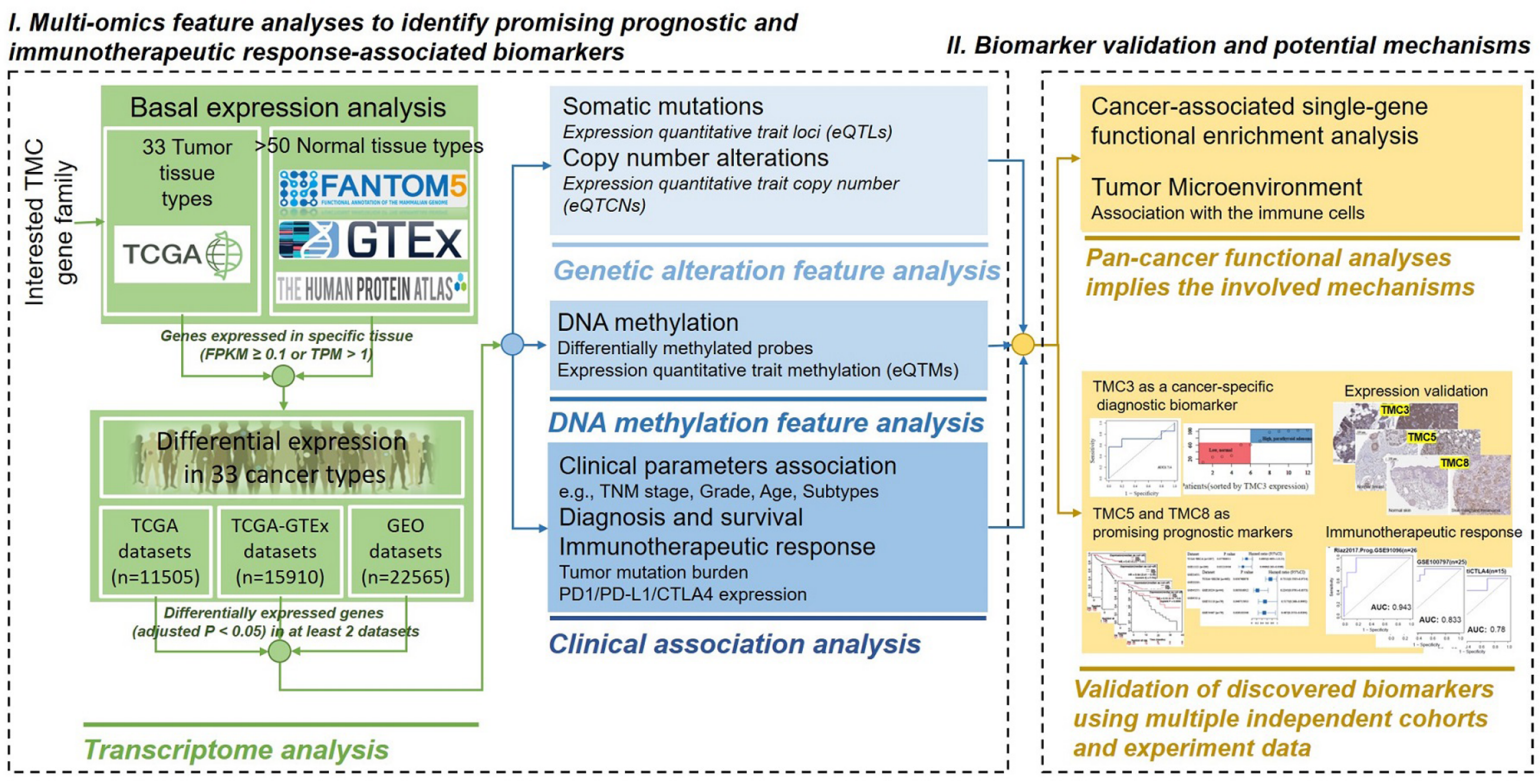
The powerful pipeline of multi-omics alteration-based clinical enrichment analysis. First, we explored the basal expression and differential expression of TMCs in >50 human tissue types and >30 cancer types. Then, the analyses of the multi-omics features of TMCs in cancers were performed. We identified prognostic and therapeutic biomarkers. Finally, we investigated the functional implication of TMCs in cancer and validated the discovered biomarkers using multiple independent cohorts and experiment data.

In this study, we performed multi-omics feature analysis to identify the link between genetic alterations of TMCs and clinical events (e.g., immunotherapeutic responses) by leveraging multi-omics big data of the same tumors. We comprehensively investigated the basal expression levels of TMC family genes in human normal tissues, and their expression dysregulations, DNA methylation, genomic mutations, and copy number alterations in human cancer samples. We predicted their involved cancer pathways and analyzed their correlation with the tumor microenvironment. We evaluated their potential value in prognosis and immunotherapeutic response in diverse human cancers by leveraging the multi-omics tumor big data (**Figure 1**).

## Results

### Basal Expression of TMC Family Genes in Human Cancer and Normal Tissues

**Figure 2:**
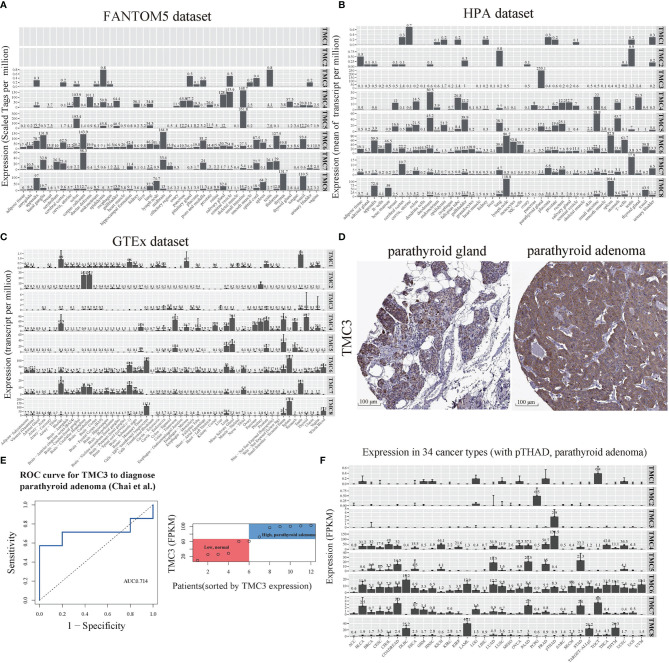
The basal expression of TMC genes in human tissues and cancers. The mRNA expression of TMCs 1-8 based on data from the **(A)** FANTOM5 database (45 tissue types), **(B)** HPA database (43 tissue types), and **(C)** GTEx database (54 tissue types). **(D)** The immunohistochemistry of TMC3 in the parathyroid gland and parathyroid adenoma. **(E)** The receiver operating characteristic (ROC) curve and the range of mRNA expression values of TMC3 in parathyroid adenoma using data from Chai et al. **(F)** The mRNA expression of TMCs 1-8 in 34 cancer types. The median expression (zeros were omitted) of each gene in each tissue/cancer type was shown. FPKM, fragment per kilobase per million. ACC, Adrenocortical carcinoma; ALL, Acute Lymphoblastic Leukemia; BLCA, Bladder urothelial carcinoma; BRCA, Breast invasive carcinoma; CESC, Cervical and endocervical cancers; CHOL, Cholangiocarcinoma; COADREAD, Colorectal adenocarcinoma; DLBC, Lymphoid Neoplasm Diffuse Large B-cell Lymphoma; ESCA, Esophageal carcinoma; GBM, Glioblastoma multiforme; HNSC, Head and Neck squamous cell carcinoma; KICH, Kidney Chromophobe; KIRC, Kidney renal clear cell carcinoma; KIRP, Kidney renal papillary cell carcinoma; LAML, Acute Myeloid Leukemia; LGG Brain Lower Grade Glioma; LIHC, Liver hepatocellular carcinoma; LUAD, Lung adenocarcinoma; LUSC Lung squamous cell carcinoma; MESO, Mesothelioma; OVCA, Ovarian serous cystadenocarcinoma; PAAD, Pancreatic adenocarcinoma; PCPG, Pheochromocytoma and Paraganglioma; PRAD, Prostate adenocarcinoma; SARC, Sarcoma; SKCM, Skin Cutaneous Melanoma; STAD, Stomach adenocarcinoma; TGCT, Testicular Germ Cell Tumors; THCA, Thyroid carcinoma; THYM, Thymoma; UCEC, Uterine Corpus Endometrial Carcinoma; UCS, Uterine Carcinosarcoma; UVM, Uveal Melanoma.

We analyzed the expression of TMC family genes in human tissues and cancer samples to investigate the role of TMC family genes in human cancers. The data from the FANTOM5, HPA, and databases showed concordant results that the TMC1 and TMC2 were almost not expressed in all analyzed tissues and cancer types (**Figures 2A**–**C**). TMC3 was specifically expressed in the parathyroid gland (**Figure 2B**). Immunohistochemistry showed that the expression of TMC3 was higher in parathyroid adenoma than that in normal parathyroid tissue (**Figure 2D**). The expression of TMC3 could be a specific classifier to distinguish the parathyroid adenoma samples and the normal parathyroid tissue (**Figures 2D, E**). However, TMC4, TMC5, TMC6, TMC7, and TMC8 were widely expressed in diverse tissue types. Further, TMC4, TMC5, TMC6, TMC7, and TMC8 were tissue-specifically and cancer-specifically expressed (**Figures 2A**–**C, F**). This reveals that they may play different roles in different cancer types.

### The TMC Family of Genes Were Deregulated and Were Affected by the Mutations, eQTM and eQTCN in Human Cancers

**Figure 3:**
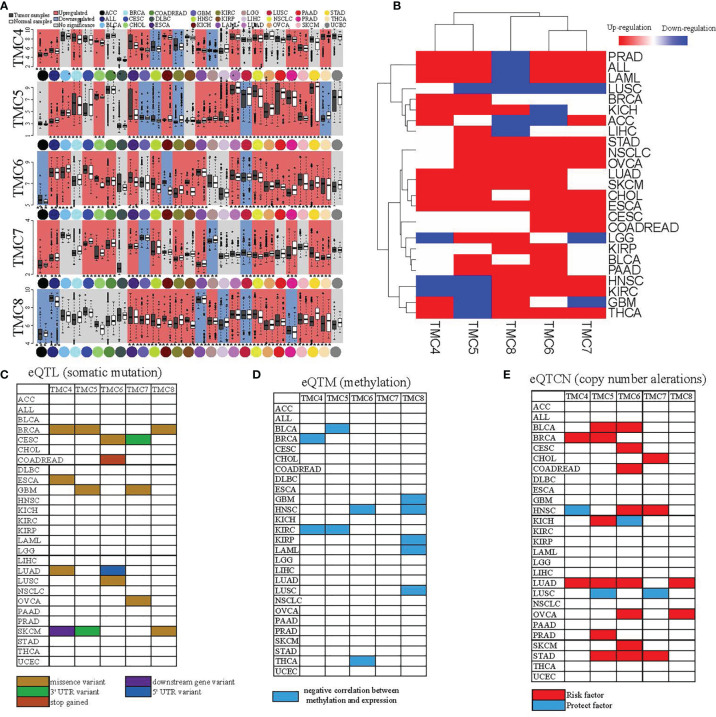
The differential expression and genomic changes of TMC genes in human cancers. **(A)** The differential expression of TMCs 4-8 in the GEO datasets. The differential expression status shown was confirmed based on TCGA datasets and the TCGA-GTEx datasets. **(B)** Hierarchical clustering of the expression status of TMCs 4-8 in diverse cancer types. The expression statuses were validated based on the three different datasets. **(C)** The expression quantitative trait loci (eQTL), **(D)** the expression quantitative trait methylation (eQTM), and **(E)** the expression quantitative trait copy number (eQTCN) of TMCs 4-8 in human cancers.

We performed differential expression analysis to determine which of the TMC genes were potentially important in human cancers based on the GDC-TCGA, GEO, and TCGA-GTEx datasets. The TMC genes were considered differentially expressed if they were detected in more than one dataset and mutually validated. The results showed that TMC4 and TMC5 have a similar expression pattern in different cancers, as do TMC6 and TMC7 (**Figure 3A**). Based on the expression of TMC genes, prostate adenocarcinoma (PRAD), acute myeloid leukemia (LAML), and acute lymphoblastic leukemia (ALL) were grouped (**Figure 3B**). However, the expression of TMC genes in lung adenocarcinoma and lung squamous carcinoma was different, indicating that the roles of TMC genes in the two lung cancer subtypes may be different (**Figure 3B**).

**Figure 4:**
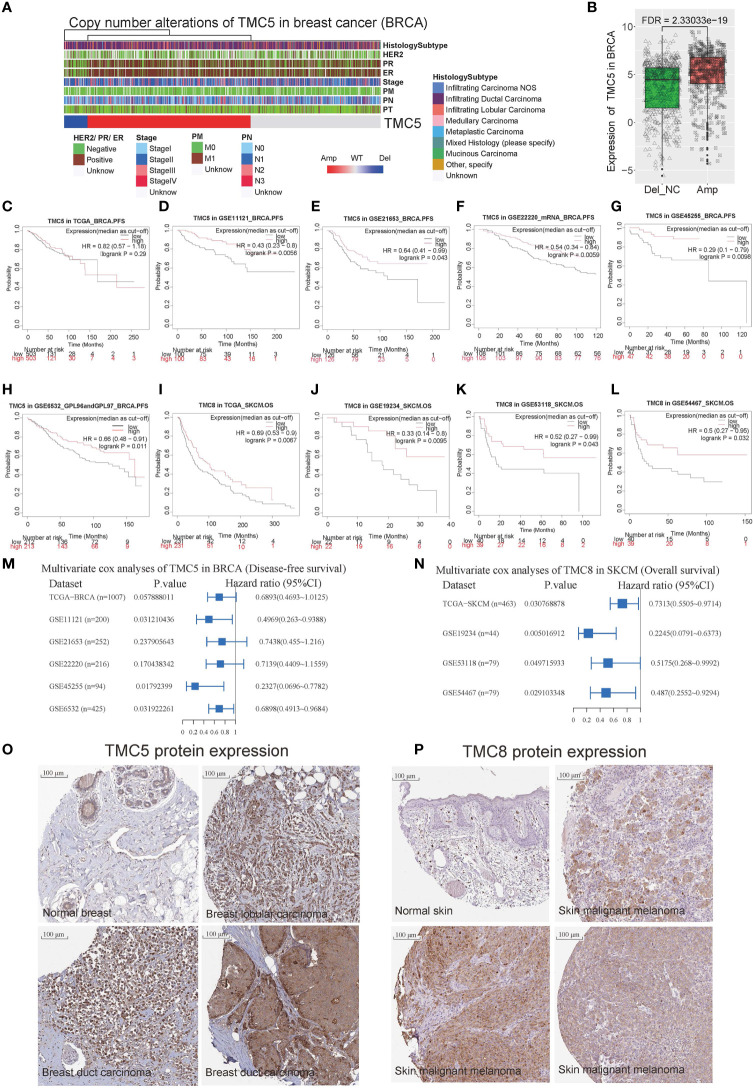
The role of TMC5 and TMC8 in breast cancer and skin melanoma. **(A)** The clinical characteristics of patients with TMC5 copy number alteration. **(B)** The expression of TMC5 between samples with and without TMC copy number amplification. The Kaplan-Meier curves of progression-free survival for TMC5 expression (the median expression as a cut-off) in breast cancer based on **(C)** TCGA-BRCA cohort, **(D)** GSE11121 cohort, **(E)** GSE21654 cohort, **(F)** GSE22220 cohort, **(G)** GSE45255 cohort, and **(H)** GSE6532 cohort. The Kaplan-Meier curve of overall survival for TMC8 expression (the median expression as a cut-off) in skin melanoma based on **(I)** TCGA-SKCM cohort, **(J)** GSE19234 cohort, **(K)** GSE53118 cohort, **(L)** and GSE54467 cohort. **(M)** The multivariate Cox regression analysis for progression-free survival of TMC5 (median expression as a cut-off) based on the six independent breast cancer cohorts. **(N)** The multivariate Cox regression analysis for overall survival of TMC8 (median expression as a cut-off) based on the four independent skin melanoma cohorts. **(O)** The immunohistochemistry of TMC5 in normal breast tissue and breast tumors. **(P)** The immunohistochemistry of TMC8 in normal skin tissue and skin melanomas.

We further investigated the DNA methylation, somatic mutations, and copy number alterations of TMC genes in human cancers to identify the cancer-associated genomic changes. The significantly mutated eQTLs of TMC family genes were mainly detected in breast cancer (**Figure 3C**). TMC8 is the most frequently affected gene by DNA methylation among the TMC family genes (**Figure 3D**). The CNAs of TMC genes were mainly found in lung adenocarcinoma, stomach adenocarcinoma, and head and neck squamous carcinoma (**Figure 3E**). Most of the CNAs are amplification. Besides, TMC5 has the highest frequency of CNAs in tumors (523/1062, 49.2%, **Figure 4A**). TMC amplifications were enriched in ER-positive breast cancer (χ^2^ test, P = 1.402e-09), PR-positive breast cancer (χ^2^ test, P = 3.489e-07), and tumor-infiltrating breast subtype (χ^2^ test, P = 0.011, infiltrating carcinoma versus non-infiltrating carcinoma). Copy number amplification upregulated the expressions of TMC5 in breast cancer (**Figure 4B**). The cancer-associated genomic changes of TMCs were listed in **Table S4**.

### The Prognostic Potential of TMC Family Genes

To investigate the prognostic potential of TMC family genes in human cancers, we performed univariate and multivariate Cox regression analyses based on the TCGA and GEO cohorts. Six independent breast cancer cohorts (TCGA-BRCA, GSE11121 (14), GSE21653 (15), GSE22220 (16), GSE45255 (17), and GSE6532 (18). Patient information is summarized in **Table S5**) reveal TMC5 was associated with disease-free survival of breast cancer (**Figures 4C**–**H**). Multivariate analyses based on TMC5 expression (median as the cut-off to divide the patients into high- or low-group) and patient clinical characteristics in the three breast cancer cohorts (GSE11121, GSE45255, GSE6532) showed that the TMC5 expression was an independent prognostic biomarker for disease-free survival in breast cancer (**Figure 4M**). Moreover, four independent skin melanoma cohorts (TCGA-SKCM cohort, GSE19234 (19), GSE53118 (20), and GSE54467 (21), **Table S6**) concordantly reveal low expression of TMC8 was related to the poor overall survival of skin melanoma (**Figures 4I**–**L**). Multivariate analyses based on TMC8 expression (median as the cut-off to divide the patients into high- or low-group) and patient clinical characteristics in the four cohorts showed that TMC8 expression was an independent prognostic biomarker for overall survival in skin melanoma (**Figure 4N**). Moreover, the protein expression of TMC5 and TMC8 in breast cancer (18 of 21 with medium/high intensity, 4 are displayed in **Figure 4O**) and skin melanoma (9 of 10 with medium/high intensity, 4 are displayed in **Figure 4P**) were respectively validated using immunohistochemistry data.

### Functions and Pathways of TMC Family Genes in Human Cancers

**Figure 5:**
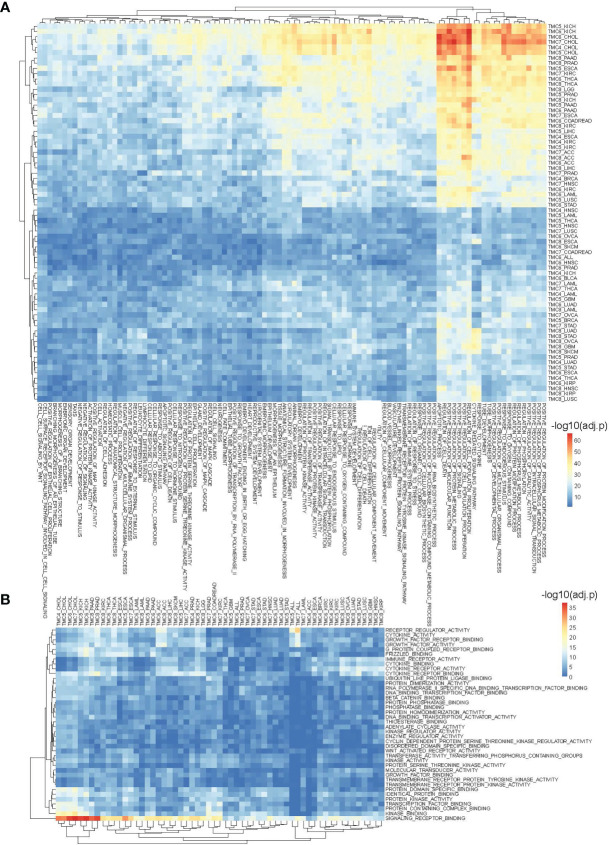
The involved biological processes and molecular functions of differentially expressed TMCs in human cancers. **(A)** The involved biological processes and **(B)** molecular functions of TMCs 4-8 in cancers. The genes significantly correlated with TMC genes in each cancer were used for functional enrichment analysis.

**Figure 6:**
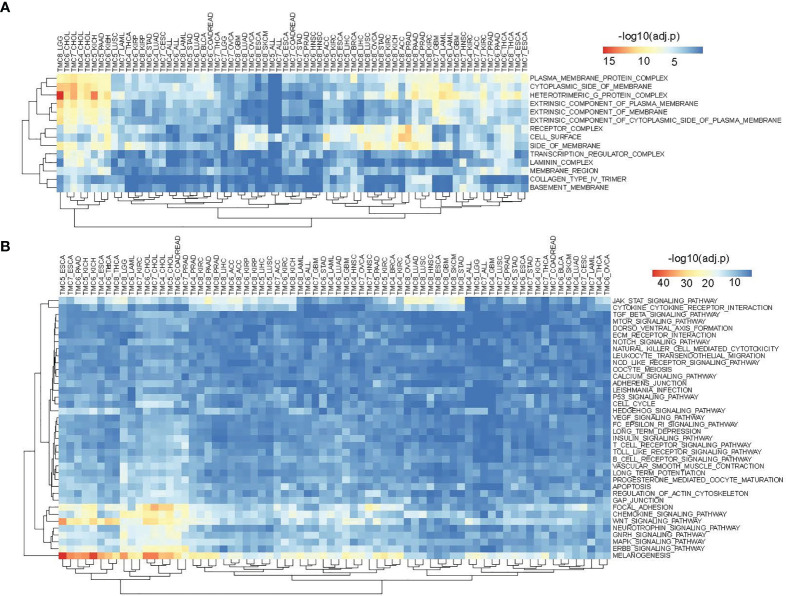
The involved cellular components and signaling pathways of differentially expressed TMCs in human cancers. **(A)** The involved cellular components of TMCs 4-8 in human cancers. **(B)** The involved KEGG pathways of TMCs 4-8 in cancers.

We performed cancer-related functional and pathway analyses to investigate the potential role of TMC genes in human cancers. For each cancer type, the involved GO functions and KEGG pathways were analyzed only if the TMC genes were differentially expressed. Results showed that the genes significantly correlated with TMC genes in each cancer type may play similar roles in human cancers. They are mainly involved in intracellular and cell-cell cellular signal transduction to regulate cell proliferation, differentiation, and apoptosis by the stimulus of the endogenous (**Figure 5A**). Consistently, the common molecular functions they are involved in are signaling receptor binding (**Figure 5B**). Unsurprisingly, the cellular components they are involved in are the plasma membrane (**Figure 6A**). TMCs may play different functions in different tumors. For example, TMCs 4-8 are involved in the regulation of cell proliferation and cell death in KICH and CHOL (FDR<10e-50), but may not be involved in these functions for HNSC and OVCA (**Figure 5A**). The pathway analysis indicated that the differentially expressed TMC genes were associated with melanogenesis and Wnt signaling (**Figure 6B**). Importantly, we noticed that the results showed that TMCs may play a role in the tumor microenvironment. Specifically, GO biological process analysis showed that deregulated TMCs were involved in immune system regulation, leukocytes/lymphocytes differentiation (**Figure 5A**). GO molecular function analysis revealed that deregulated TMCs were involved in immune receptor activities (**Figure 5B**). Pathway analysis also showed that deregulated TMCs were associated with T cell/B cell receptor activity, NK cell-mediated cytotoxicity, and leukocyte transendothelial migration (**Figure 6B**). It is known that leukocyte transendothelial migration is indispensable to initialize the innate or adaptive immune response. Together, these results indicated the critical role of TMCs in the tumor microenvironment of diver cancers.

### The Role of TMC Genes in the Tumor Microenvironment and Immunotherapy Response of Human Cancers

**Figure 7:**
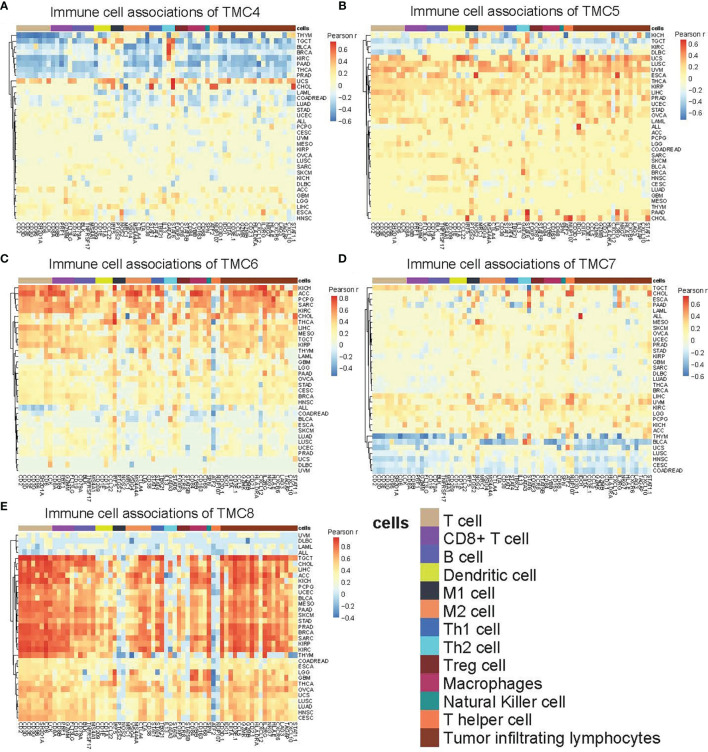
The immune-cell relevance of TMCs in human cancers. The relevance between 13 types of immune cells and **(A)** TMC4, **(B)** TMC5, **(C)** TMC6, **(D)** TMC7, and **(E)** TMC8 in 33 human cancer types. For better visualization, the cells with Pearson correlation coefficients r were set to zero if the Benjamini-Hochberg adjusted P value > 0.01.

**Figure 8:**
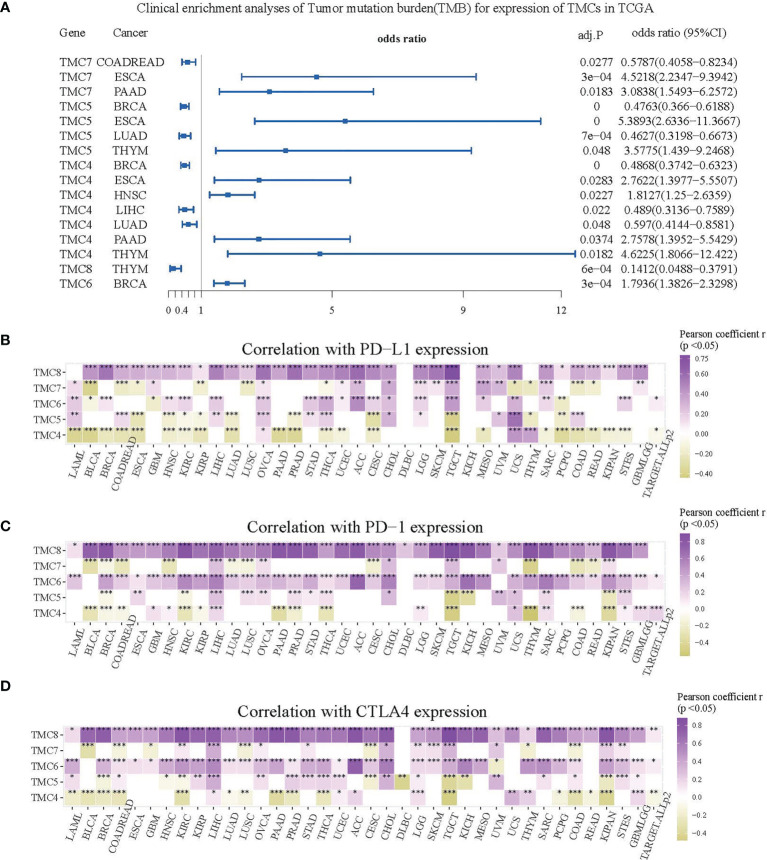
The role of TMCs in immunotherapeutic response. **(A)** The forest plot for the clinical enrichment analyses of tumor mutation burden based on the expression of TMCs in the TCGA datasets. **(B–D)** The expression correlation between TMCs and PD-1/PD-L1/CTLA4 in 33 cancer types. Only cells with P < 0.05 were shown in color. *P < 0.05, **P < 0.01, ***P < 0.001.

**Figure 9:**
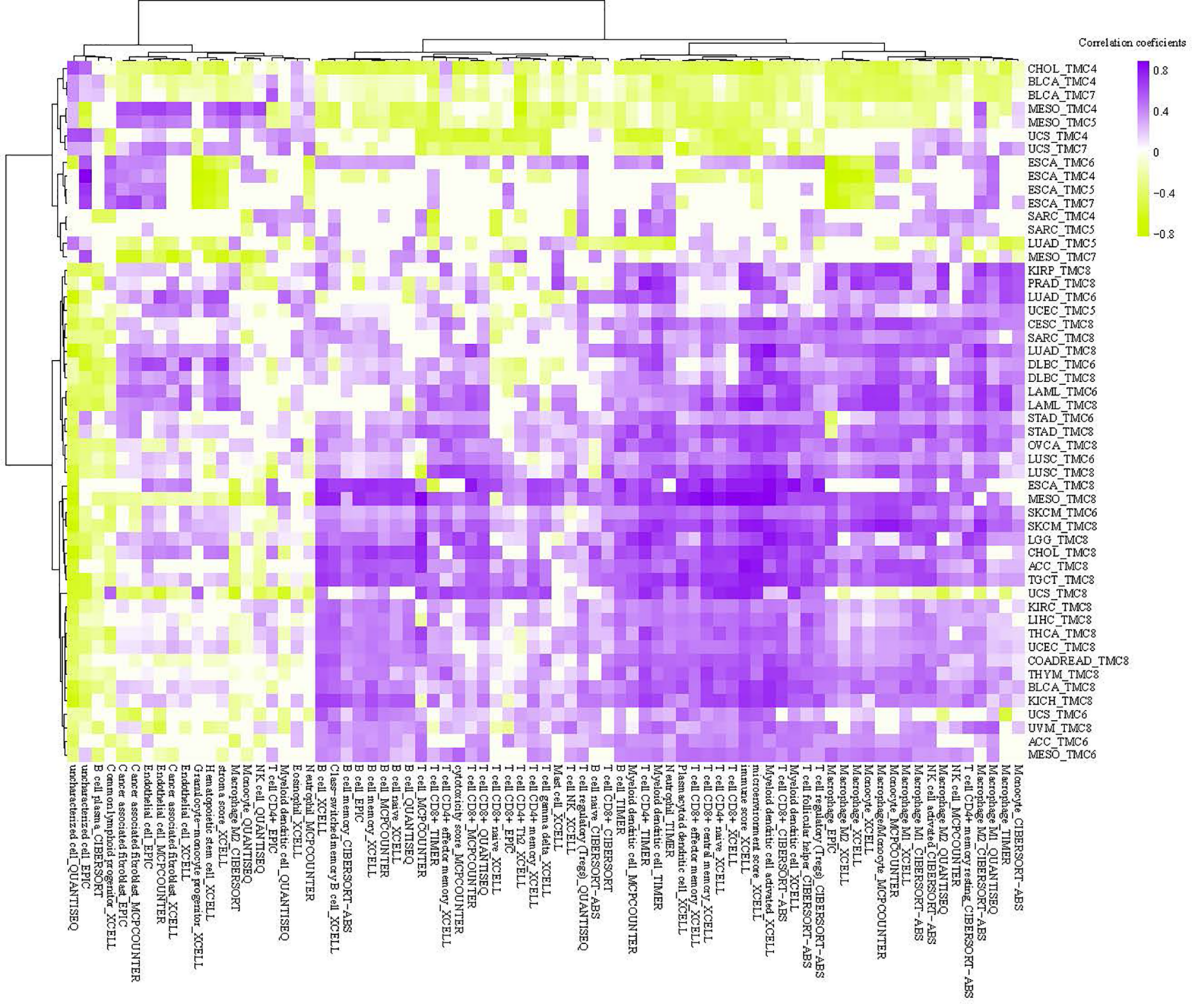
The tumor microenvironment analysis of TMCs in cancer. The correlation between single sample tumor-infiltrating estimation scores (estimated based on CIBERSORT, EPIC, MCP-counter, quanTIseq, xCell, and TIMER methods) and expression of TMCs in cancer.

We investigated the correlation between immune cells and TMC genes in human cancers (**Figure 7**). Results showed that the TMC6 and TMC8 showed higher similarity in tumor immune cells (**Figures 7C, E**). Different from the classification based on amino acid sequence homology, TMC5, and TMC6 belong to the same subfamily (subfamily 2) (5), but they showed opposite relationships with immune cells in cancers (**Figures 7B, C**). Notably, TMC8 was highly correlated with T/CD8+ T cells, B cells, and tumor-infiltrating lymphocytes in 29 cancers (**Figure 7E**), while TMCs 4 and 7 were negatively correlated with these cells in some of the cancers such as THYM and BLCA (**Figures 7A, D**). We further performed the correlation between the expression of TMCs and tumor mutation burdens and PD-L1 expression to evaluate the potential immunotherapeutic response. Results showed that TMC5 was significantly associated with tumor mutation burdens in esophagus carcinoma, lung adenocarcinoma, and breast cancer (**Figure 8A**). TMCs 4-8 were associated with PD-1/PD-L1/CTLA4 expression in diverse cancer types, such as breast cancer and skin melanoma (**Figures 8B**–**D**). Analysis of computed tumor immune infiltrating estimation score (such as CIBERSORT and xCell algorithm) also revealed that the TMCs were significantly associated with the infiltrating immune cells (**Figure 9**, BH-adjusted P<0.01). For example, TMC6 and TMC8 were highly positively correlated with macrophages, B cells, and T cells in most cancer types, while TMC4, TMC5, and TMC7 were negatively correlated with those cells in diverse cancer types (**Figure 9**).

### The Predictive Value of TMCs in Immunotherapeutic Clinical Benefits

**Figure 10:**
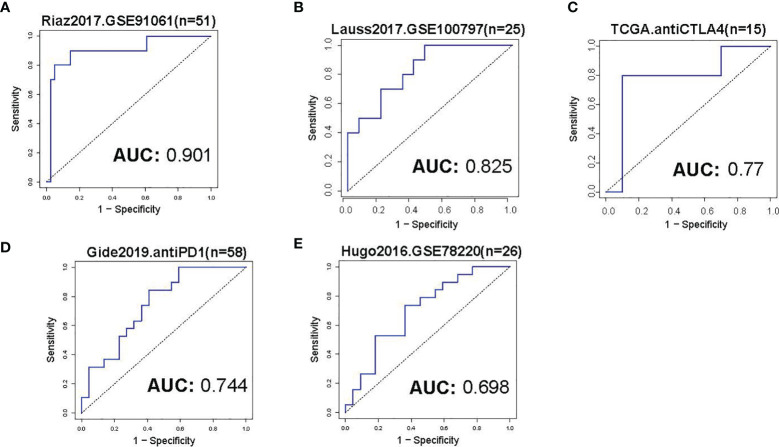
Evaluation of immunotherapeutic response. **(A)** The ROC curve of IRP score in prediction of immunotherapeutic response of skin melanoma in the training set. The median value of the IRP score was used as a cut-off to divide the patients into low- and high groups. **(B–E)** The ROC curves of IRP scores in the prediction of immunotherapeutic response of skin melanoma in the validation sets. The immunotherapy information of the analyzed datasets was shown in **Table 1**.

The cohorts used to evaluate the predictive value of TMCs in immunotherapeutic clinical benefits were summarized in **Table 1**. The Riaz2017.GSE91061 cohort (n=51) was used as training set and the other cohorts were used as validation sets. We developed an IRP score based on the expression of TMCs 4-8 signature to predict whether patients have clinical benefit. The median value of the IPR score was used as the cut-off to divided the patients into high and low groups. Results showed that the IRP score had a high sensitivity and specificity in the training set (P = 0.017, odds ratio = 2.732, 95% confidence interval = 1.267-7.342, AUC=0.901, **Figure 10A**), Lauss2017.GSE100797 cohort (P = 0.028, odds ratio = 2.309, 95% confidence interval = 1.222-11.825, AUC=0.825, **Figure 10B**), and TCGA.antiCTLA4 set (P = 0.031, odds ratio = 2.145, 95% confidence interval = 1.192-8.432, AUC=0.77, **Figure 10C**). It also showed a high performance in Gide2019.antiPD1/antiCTLA4 cohort (P = 0.036, odds ratio = 2.007, 95% confidence interval = 1.084 -9.863, AUC=0.744, **Figure 10D**), and Hugo2016.GSE78220 (P = 0.044, odds ratio = 1.809, 95% confidence interval = 1.034-7.432, AUC=0.698, **Figure 10E**). Thus, all cohorts concordantly showed that a higher IRP score predicts a higher immunotherapeutic response.

**Table 1:** Skin melanoma cohorts for analysis of immunotherapeutic clinical benefits.

Cohort	No. of patients	Therapy
TCGA-SKCM	15	anti-CTLA4
Gide2019.antiPD1/antiCTLA4	58	anti-PD1/anti-CTLA4
Riaz2017.GSE91061	51	anti-PD1
Lauss2017.GSE100797	25	Adoptive T cell therapy
Hugo2016.GSE78220	26	anti-PD1

## Discussion

Growing evidence showed that TMC channels may play an important role in cancer and may have clinical value. In the present study, by integrating the multi-omics pan-cancer big data, we systematically analyzed the basal expression levels of TMC family genes in human normal tissues, and their expression dysregulations, DNA methylation, genomic changes, involved cancerous pathways, their correlation with the immune cells and immunotherapeutic response, and their clinical potential in human cancer samples. We show that the TMC genes were tissue-specifically expressed. TMCs were differentially expressed in many cancer types and may be affected by DNA methylation disorders and genomic alterations. We found that TMCs were significantly correlated with patient survival of diverse cancers. For example, six cohorts validated that lower expression of TMC5 was associated with a higher risk of post-treatment relapse of breast cancer. Also, four cohorts showed that lower expression TMC8 was associated with the poorer overall survival of skin melanoma. Functional and pathway analyses indicated that TMCs play a critical role in the tumor microenvironment. Furthermore, TMCs were commonly associated with TMB, and expression of PD-1/PD-L1/CTLA4, which implied that TMCs may be of potential value in the prediction of immunotherapeutic response. Finally, we developed a novel signature based on TMCs 4-8. The higher level of the signature predicts the higher immunotherapeutic response.

The previous evidence showed that TMCs 1-3 are expressed at low levels in human tissues, whereas TMCs 4-8 are expressed at higher levels in multiple tissues. Here, we analyzed the basal expression levels of TMC genes in ~50 tissue types and >30 cancer types. The results further confirmed that TMCs 1-3 were almost not expressed in all investigated normal tissues and cancers, while the TMCs 4-8 were widely expressed in diverse tissues and cancers. Surprisingly, we discover that TMC3 was specifically expressed in parathyroid tissue and could distinguish the parathyroid adenoma (higher TMC3 levels) and normal parathyroid glands (lower TMC3 levels). Moreover, immunochemistry data further validated the higher expression level of TMC3 in parathyroid adenoma compared with parathyroid glands. Currently, the preoperative diagnosis of parathyroid adenoma is difficult and mainly depends on clinical parameters, such as clinical symptoms, imaging, and parathyroid gland weight (22, 23). The molecular biomarker may improve the diagnosis of parathyroid adenoma.

We observed the frequent dysregulation of TMCs 4-8 in human cancers. The dysregulations of TMCs were significantly correlated with overall survival and relapse, indicating that TMCs may play an important role in cancer biology. The DNA methylation and genomic changes can affect the expression of TMC genes in cancer, thus the differential DNA methylation and genomic mutation and CNVs were analyzed. We identified 16 common mutations, 11 differentially methylated CpGs, and 25 copy number alterations of TMC genes in cancers, such as TMC5 amplification, were frequently detected in breast cancer (523/1062, 49.2% of patients). These epigenomic and genomic changes may be responsible for the cancer-specific dysregulation of TMC genes in cancer. Moreover, the cancer-specific dysregulation of TMC genes suggests that TMC genes may play different roles in different cancer types. To investigate how the TMCs play a role in cancer progression, we analyzed their functions and pathways. Results indicated that TMCs were associated with the tumor microenvironment. We found that the relationship between TMC genes and immune cells varies in different cancers. Interestingly, in 29 types of solid tumors, TMC8 showed a high positive correlation with T cells, B cells, and tumor-infiltrating lymphocytes, while TMCs 4 and 7, which belonged to the same subfamily with TMC8 (5), showed the opposite relationship with immune cells. It suggested that TMC8 and TMCs 4 and 7 may belong to different functional subfamilies in cancer. Currently, many robust tools can evaluate the immune cell proportions, such as CIBERSORT, xCell, and ImmuCellAI (24). Multiple tools were applied and the evidence of single sample infiltrating estimations further validated the association between TMCs and tumor microenvironment. Because of the significant association between TMCs and tumor microenvironment, TMB, and PD-1/PD-L1/CTLA4, we hypothesis that TMCs may have predictive value in immunotherapeutic response. We focussed on the evaluation of the predictive role of TMCs in skin melanoma. We used the Riaz.2017 dataset as the training set to fit the model for the prediction of immunotherapy. Results showed that it had high sensitivity and specificity (AUC >0.9). We keep in mind that the anti-PD1 therapy was applied to the patients in the training dataset. Thus further validation using two independent cohorts of anti-PD1 therapy (Gide2019 dataset and Hugo2016 dataset, **Table 1**) was performed. It confirmed the good performance of TMCs signature in the prediction of anti-PD1 immunotherapy response (AUCs >0.7). The performance of the TMCs signature in the Hugo2016 dataset was relatively low, probably because of the small sample size. Moreover, we further investigated the predictive value of TMC signatures in other types of immunotherapy. Surprisingly, the performance of the TMC signatures in anti-CTLA4 and adoptive T cell therapy (AUCs >0.75) is promising, implying that TMCs might serve as a more generalized tool for the prediction of immunotherapeutic responses. Taken together, all cohorts concordantly showed that a higher IRP score predicts a higher immunotherapeutic response.

## Conclusions

In conclusion, we show that TMC5 is an independent prognostic marker for post-treatment relapse of breast cancer. Also, four cohorts showed that TMC8 is an independent prognostic marker for the overall survival of skin melanoma. A novel five-gene signature (i.e., TMCs 4-8) was established for IRP in skin melanoma. Patients with higher IRP scores are predicted to have higher immunotherapeutic clinical benefits. These data provide a landscape of the functional role of TMC channels in cancer and may provide new biomarkers for the prognosis and immunotherapeutic response of breast cancer and skin melanoma. Our analysis pipeline may be utilized for multi-omics feature investigations in future studies encompassing other gene families, or gene signatures, which will uncover more clinical-associated omics changes in cancer.

## Materials and Methods

### Cancer Multi-Omics Data

The gene expression data of human tissues were downloaded from the FANTOM5 (45 tissue types) (25), Human Protein Atlas (HPA, 43 tissue types) (26), and GTEx databases (54 tissue types) (27). The DNA methylation data, somatic mutation data, copy number alteration (CNA) data, mRNA expression, and patient clinical information from the TARGET and Cancer Genome Atlas (TCGA) projects (33 cancer types, https://portal.gdc.cancer.gov/) were also downloaded. The expression data of the TCGA-GTEx dataset were downloaded from the UCSC Xena (https://xena.ucsc.edu/). This dataset was generated based on the re-computation of raw RNA-seq data from TCGA and GTEx projects by the UCSC Xena project and was widely used as affiliate evidence to compare tumor-normal expression differences. We further integrated the expression array of GPL570 (Affymetrix Human Genome U133 Plus 2.0 Array) of 27 cancer types from the Gene Expression Omnibus (GEO) database. Also, we integrated the DNA methylation array of GPL13534 (Illumina HumanMethylation450 BeadChip) for 27 cancer types from the TCGA and GEO databases. The multi-omics data were preprocessed as described before (2). Precisely, the raw.CEL expression data of the GPL570 array from GEO were downloaded and preprocessed using the affy package. The expression matrix of each cancer type was quantile normalized for downstream analyses. The raw.idat methylation data of the GPL13534 array from GEO and TCGA were preprocessed using the minfi package (28) and were integrated for each cancer type. The datasets used in this study are listed in **Tables S1**–**S3**.

### Expression, Mutation, DNA Methylation, and Copy Number Alteration Analyses

The basal expressions of TMC family genes were investigated based on FANTOM5, HPA, and GTEx datasets. Genes were considered not expressed if protein-coding transcript per million (pTPM) expression of FANTOM5 < 1, scaled tags per million expressions of HPA < 1, transcript per million (TPM) of GTEx < 1, or fragments per kilobase of transcript per million fragments mapped (FPKM) of TCGA < 0.1.

As TMC1, TMC2, and TMC3 were not expressed in almost all analyzed tissues, the differential analyses, mutation, and copy number alteration analyses were only performed in 27 cancer types. The differential methylation analysis and differential expression analysis for microarray data were performed based on the limma package in R software. The expression probes and methylation probes with Benjamini-Hochberg adjusted P value <0.05 were retained. The CNA analysis was based on GISTIC2.0 software (29). The somatic mutation analysis for each cancer was performed based on the MAF files from the GDC data portal (https://portal.gdc.cancer.gov/). The somatic mutations with frequency >= 0.05 of each cancer were considered significant mutations. The expression quantitative trait loci (eQTL) data of >50 human tissues were downloaded from the GTEx database. The genes with somatic mutations identified in >5% of patients of specific cancer were considered as significantly mutated genes. We identified the expression quantitative trait methylation (eQTM) and expression quantitative trait copy number (eQTCN) using paired expression-methylation or expression-copy number data based on the methods described before. The eQTMs and eQTCNs with Benjamini-Hochberg adjusted P value <0.05 were retained.

### Functional and Pathway Analyses of TMC Family Genes in Human Cancers

The Gene Ontology (GO) functional analysis and Kyoto Encyclopedia of Genes and Genomes (KEGG) pathway analysis were performed based on the genes significantly correlated with TMC genes in each cancer type. The expressions of gene pairs with Pearson correlation coefficients |r| ≥0.3 and Benjamini-Hochberg adjusted P values <0.01 were considered significantly associated. The 531 genes in the “pathway in cancer” super pathway of PathCards (https://pathcards.genecards.org/) were considered commonly cancer-associated genes and were used to perform gene expression correlation analyses. The enriched GO terms and pathways with Benjamini-Hochberg adjusted P value <0.01 were considered statistically significant.

### Immune Cell/Infiltrating Analysis and Clinical Enrichment Analysis of TMC Family Genes

To analyze the correlation between TMC genes and immune cells in each cancer type, we performed the Pearson correlation analyses based on the expression of TMC genes and the marker genes of the immune cells (T cells, CD8+ T cell, B cell, dendritic cell, M1/M2 cell, Th1/Th2 cell, Treg cell, Macrophages, Natural killer cell, T helper cell, and Tumor-infiltrating lymphocytes) (30). The marker genes of the immune cells with Benjamini-Hochberg adjusted P value <0.01 were considered statistically significant. The tumor mutation burdens (TMB) of each sample called by MUSE software from the TCGA datasets were downloaded from the GDC data portal. Fisher’s exact test was used to test whether the high-expression group (median as cut-off) were significantly enriched in the high-TMB group (median as cut-off). The correlation between TMCs expression and PD-1/PD-L1/CTLA4 expression was also analyzed. The tumor-infiltrating estimation score of each cell type for each sample was calculated based on CIBERSORT (31), EPIC (32), MCP-counter (33), xCell (34), quanTIseq (35), and TIMER (36) methods. Pearson correlations between infiltrating cell types and TMCs in 33 cancer types were performed to investigate the role of TMCs in tumor-infiltrating. The gene-cell pairs with BH-corrected P values < 0.05 were considered significant.

### The Predictive Value of TMCs in Immunotherapy Response

The transcriptome data and corresponding clinical information of seven skin melanoma cohorts (TCGA-SKCM [n=15], Gide2019.antiPD/1antiCTLA4 [n=58] (37), Riaz2017. GSE91061 [n=51] (38), Lauss2017.GSE100797 [n=25] (39), and Hugo2016.GSE78220 [n=26] (40)) downloaded from the TIDE database (41) were used to evaluate the predictive value of TMCs in immunotherapeutic clinical benefits. Patients were treated with adoptive T cell therapy, anti-PD1, and/or anti-CTLA4 therapy. The treatment information of the seven cohorts was summarized in **Table 1**. After immunotherapy, patients with complete remission, partial remission (the RECIST criteria), or otherwise with an Overall Survival (OS) time of more than 2 years were considered to have clinical benefits. The logistic regression was performed based on the high/low level of an immunotherapy response predictive (IRP) score calculated based on the expressions of TMCs 4-8 as the following formula:


Equation 1 $$IRP\;score=\Sigma_{i=1}^{n}(coef_{i}*\exp_{i})$$

where n is the number of TMCs 4-8 (i.e., n=5) in the model, *exp_i_* is the expression level of *TMC_i_* and *coef_i_* is the estimated regression coefficient of *TMC_i_* in the multivariate logistic regression model. The median IPR score was used as a cut-off to divide the patients in the training (Riaz2017. GSE91061) cohort and validation cohorts into low-risk and high-risk groups. The Receiver Operative Curve (ROC) and Area Under Curve (AUC) were used to evaluate the sensitivity and specificity of the classifier. The 5-fold cross-validation was used to avoid overfitting.

### Cox Regression Analysis

The univariate Cox regression analyses were performed based on the expression of TMC family genes in cancers. The genes with BH-adjusted P value <0.05 were considered as candidate variables and were subjected to the multivariate Cox regression model. The multivariate analyses were performed based on the TMC gene expression and patient clinical information, such as TNM stage, grade, and age. The Cox regression analysis was performed using *survival* and *survplot* packages in R software. Genes with the log-rank P value <0.05 were considered statistically significant.

### Immunohistochemistry Data of HPA

The immunohistochemistry data from HPA (26) were used to validate the protein expression levels of TMC3, TMC5, and TMC8 in human normal tissues and tumors. The protein expressions of TMC3 (antibody serial number: HPA040510) were analyzed in the normal parathyroid gland (1 patient sample was analyzed, i.e., patient 5745) and parathyroid adenoma (1 patient sample was analyzed, i.e., patient 3176). The protein expressions of TMC5 (antibody serial number: HPA040810 and HPA042037) were analyzed in normal breast (patient 2773), breast with lobular carcinoma (patient 3546), and breast with duct carcinoma (patient 1910 and patient 2428). A total of 21 patient samples of breast cancer were analyzed. The protein expressions of TMC8 (antibody serial number: HPA054429) were analyzed in normal skin (patient 2773), and skin with malignant melanoma (patient 2534, patient 3060, and patient 4023). A total of 10 patient samples of skin melanoma were analyzed.

## Data Availability Statement

The original contributions presented in the study are included in the article/**Supplementary Material**. Further inquiries can be directed to the corresponding authors.

## Author Contributions

Conceptualization, JS, FS, and LR. Methodology, JS and LR. Software, JS. Resources, XL. Data curation, XS and YT. Writing–original draft preparation, JS. Writing–review and editing, JS. Visualization, JS. Supervision, LR. Project administration, LR. Funding acquisition, LR and FS. All authors have read and agreed to the published version of the manuscript.

## Funding

This work was supported by grants partly from the Smart Medicine Project of Chongqing Medical University (Grant no. ZHYX2019015), the Natural Science Foundation of Chongqing in China (Grant no. cstc2018jcyjAX0019 to LR), the Chongqing Basic Science and Frontier Technology Re-search Project (Grant no. cstc2018jcyjAX0199 to FS), the Chongqing Natural Science Foundation (Grant no. cstc2019jcyjmsxmX0259 to FS), and the Key Project of Application Development Plan of Chongqing (Grant no. cstc2018jscx-mszdX0031 to FS).

## Conflict of Interest

The authors declare that the research was conducted in the absence of any commercial or financial relationships that could be construed as a potential conflict of interest.

## Publisher’s Note

All claims expressed in this article are solely those of the authors and do not necessarily represent those of their affiliated organizations, or those of the publisher, the editors and the reviewers. Any product that may be evaluated in this article, or claim that may be made by its manufacturer, is not guaranteed or endorsed by the publisher.
